# Voluntary running reduces loss of PTEN-deficient Purkinje cells, modulates their metabolic signaling, and improves motor deficits in mice

**DOI:** 10.3389/fncel.2026.1865302

**Published:** 2026-07-22

**Authors:** Siena R. Brazier, Kathryn L. Ditrano, David S. Betancourt-Trompa, Grace A. Belt, Olivia R. Barbarini, Ursula M. Pena, Izabella M. Espinal-San Miguel, Ana V. Rodriguez, Carly N. Immerman, Cameron J. Petrelli, Reagan R. Dennett, Rohan Reddy, Daniel J. Greenspan, Lindsay J. Walsh, Connor Enestvedt, Campbell M. Bridges, Pamela A. Snodgrass-Belt, Ileana Soto

**Affiliations:** 1 Department of Biology, Providence College, Providence, RI, United States; 2 Department of Biology, Brown University, Providence, RI, United States; 3 Department of Biology, Binghamton University, Binghamton, NY, United States

**Keywords:** AMPK, cerebellum, microglia, mTORC1, *Pten*

## Abstract

Dysregulation of the PTEN-mTORC1 signaling pathway disrupts cellular metabolism and contributes to neurodevelopmental disorders, including autism spectrum disorder (ASD). In cerebellar Purkinje cells (PCs), PTEN deficiency leads to hyperactivation of mTORC1, impaired energy homeostasis, and progressive neuronal degeneration. Given that physical exercise is a potent modulator of metabolic signaling, we investigated whether voluntary running could restore metabolic balance and ameliorate cellular and behavioral deficits in a mouse model of PC-specific PTEN deficiency. Using *Pten* conditional knockout (cKO) mice, we evaluated the effects of voluntary running on motor coordination, metabolic signaling, neuronal morphology and preservation, and social and non-social behaviors. Behavioral analyses revealed that running significantly improved motor performance in *Pten* cKO mice. At the cellular level, voluntary running increased phosphorylated AMP-activated protein kinase (pAMPK) and restored mitochondrial and lysosomal content in PC dendrites from *Pten* cKO mice. Unexpectedly, running further enhanced mTORC1 activity in *Pten* cKO mice, as indicated by increased pS6R immunoreactivity, suggesting a complex interplay between anabolic and catabolic pathways. Behavioral analyses further revealed that voluntary running reduced sex-dependent differences in social and non-social behaviors observed in sedentary *Pten* cKO mice. Despite persistent dendritic hypertrophy and synaptic VGLUT2 alterations, running reduced PC loss and partially normalized microglial morphology and activation. Together, these findings demonstrate that voluntary running improves metabolic homeostasis and neuronal preservation in *Pten* cKO mice, even in the presence of sustained mTORC1 activation. Our results highlight the therapeutic potential of physical activity in modulating metabolic pathways and mitigating neurodevelopmental pathology.

## 1 Introduction

The phosphatase and tensin homolog (PTEN) is a key negative regulator of phosphatidylinositol-3-kinase/protein kinase B (PI3K/AKT) signaling. By inhibiting PI3K/AKT-dependent activation of mammalian target of rapamycin complex 1 (mTORC1), PTEN regulates cell growth and metabolism throughout development and adulthood. Consequently, reduced PTEN signaling promotes cellular overgrowth, proliferation, and tumor formation (Carracedo and Pandolfi, 2008). Haploinsufficiency of the *Pten* gene is also associated with the onset of various neurological and neurodevelopmental conditions. PTEN germline suppression is associated with autism spectrum disorder (ASD) and intellectual disability (Hollander et al., 2011). Furthermore, PTEN-ASD is associated with macrocephaly, a phenotype observed in approximately 20% of children with autism, suggesting shared molecular and cellular mechanisms within this ASD subgroup (Currey et al., 2025).

In neurons, PTEN deficiency induces the hyperactivation of mTORC1, causing the hypertrophy of neuronal soma, dendrites, and axons during postnatal development and adulthood, supporting the contribution of PTEN insufficiency to neurodevelopmental defects and functional deficits (Kwon et al., 2006; Cupolillo et al., 2016; Tariq et al., 2022; Walsh et al., 2025). However, we previously found that dysfunction of the PTEN-PI3K/AKT-mTORC1 pathway in PTEN deficient Purkinje cells (PCs) also promotes metabolic deficits that include dysregulated activation of mTORC1 and its downstream ribosomal protein S6 (S6R), reduced AMP-activated protein kinase (AMPK) activity, and decreased total mitochondrial and lysosomal volume in dendrites (Walsh et al., 2025). Like PTEN (Feng et al., 2021), AMPK is a well-known energy sensor that inhibits mTORC1 to balance the activation of anabolic and catabolic pathways and promotes mitochondria biogenesis (Chaube et al., 2015). However, hyperactivation of mTORC1 can also inhibit AMPK leading to extended activation of the anabolic pathway and presumably the depletion of energy (Ling et al., 2020). *Ex vivo* treatment of PTEN deficient PCs with the AMPK activator AICAR (5-aminoimidazole-4-carboxamide-1-beta-D-ribofuranoside) increases their total volume of dendritic mitochondria and lysosomes (Walsh et al., 2025), supporting the role of AMPK in mitochondria biogenesis and energy restoration during metabolic stress and starvation (Hardie and Carling, 1997; Chaube et al., 2015; Herzig and Shaw, 2018).

Because physical exercise can activate AMPK in the brain (Huang et al., 2019; Feng et al., 2023) and promote PCs resilience during pathological conditions (Yun et al., 2014; Jin et al., 2023), we hypothesized that voluntary running would ameliorate neuropathological and behavioral abnormalities caused by PTEN deficiency through AMPK activation.

## 2 Material and methods

### 2.1 Animals

All animal procedures were approved by the Providence College Institutional Animal Care and Use Committee (IACUC protocols 2022–05 and 2025–04) and conducted in accordance with NIH guidelines and ARRIVE recommendations. Mice were housed under standard conditions (22 ± 2 °C; 49 ± 2% humidity; 12-h light/dark cycle) with food and water available *ad libitum*. To generate Purkinje cell-specific *Pten*-deficient mice, *Pten*^flox/flox^ mice were crossed with Pcp2-Cre mice (The Jackson Laboratory; stock numbers 006440 and 004146). Cre-positive *Pten* wild-type mice served as controls. Both sexes were included. Sample sizes were based on previous studies using this model (Cupolillo et al., 2016; Walsh et al., 2025) and ranged from 4–7 mice for histological analyses and 8–11 mice/group/sex for behavioral studies. Animal numbers were selected to balance statistical rigor and minimization of animal use. A total of 60 mice were used.

### 2.2 Mouse voluntary exercise experimental design and protocol

**Figure 1:**
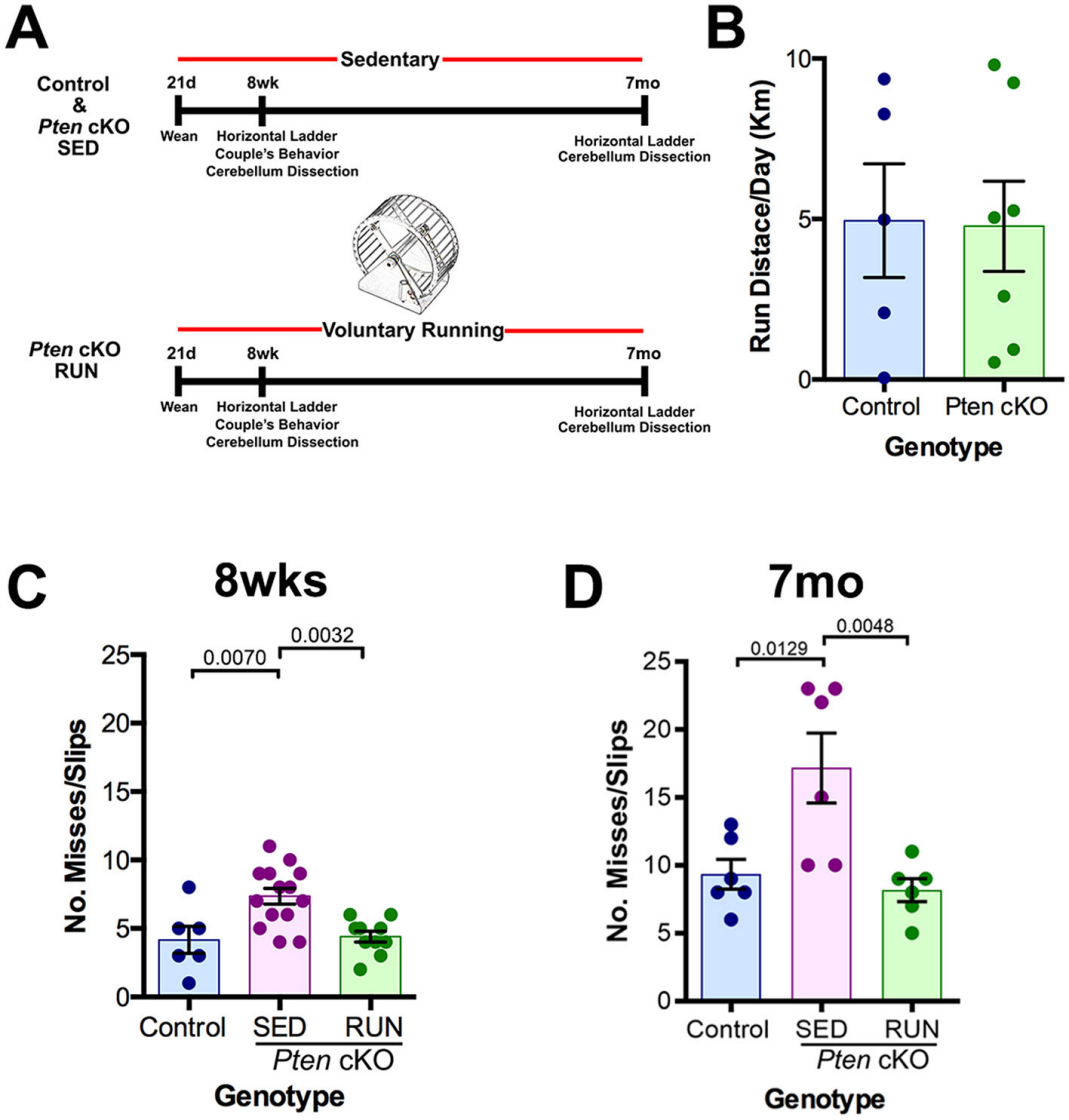
Voluntary running improves motor deficits in *Pten* cKO mice. **(A)** Experimental design to test effects of voluntary running in *Pten* cKO mice. **(B)** Average of running distance/day for control and *Pten* cKO mice demonstrating no differences between groups (*n* = 5–7 mice). **(C)** Total number of mises/slips in the ladder rung task by 8 weeks control (*n* = 6 mice), *Pten* cKO SED (*n* = 14 mice), and *Pten* cKO RUN (*n* = 10 mice). **(D)** Total number of mises/slips in the ladder rung task by 7 month control, *Pten* cKO SED, and *Pten* cKO RUN (*n* = 6 mice per group). Data presented as mean ± SEM; One Way ANOVA with *post-hoc* Tukey: **(C)** *p* = 0.001; **(D)** *p* = 0.0035.

To evaluate the effects of voluntary running, sedentary control (Cre?; *Pten* WT), sedentary *Pten* cKO (SED), and runner *Pten* cKO (RUN) mice were studied. RUN mice had access to a running wheel from postnatal day 21 until analysis (Figure 1A). Social and non-social behaviors were assessed at 8 weeks, while horizontal ladder testing and cerebellar analyses were performed at 8 weeks and 7 months to evaluate motor coordination and PC pathology.

Weaned 21-day-old mouse pups were housed in cages equipped with running wheels, either individually or in same-sex groups of up to three animals. To quantify voluntary wheel-running activity in 5-month-old *Pten* cKO and Cre-positive control mice, animals were singly housed in running wheel cages and monitored over several weeks. Wheel rotations were recorded via axle-mounted mechanical switches that registered one count per revolution.

Data were collected using ClockLab Data Collection software and analyzed with ClockLab Analysis (ActiMetrics). Mice were maintained under a standard 12-h light/12-h dark cycle to establish baseline circadian activity patterns. For distance calculations, data from days 134–148 were used. The total number of wheel revolutions per 24-h period was averaged across seven consecutive days.

Distance traveled was calculated by multiplying the total number of daily revolutions by the circumference of the running wheel. Distances were converted from centimeters to kilometers, and average daily distance per mouse was reported.

### 2.3 Ladder rung walking task

The ladder rung walking test was performed as previously reported (Walsh et al., 2025). The test consisted of mice traversing sections with 1 cm spacing at the start and end of the test but encountering a challenging 22 cm central stretch with 2 cm spacing between rungs where slips and misses were quantified. The task was performed three times for each mouse and the performance data from the three trials was summed.

### 2.4 Social and non-social interactions analysis

Mice were housed in standard conditions and allowed to acclimate their environment before testing. Heterosexual couples of 8 weeks Control, *Pten* cKO SED, or *Pten* cKO RUN mice were placed in an empty new cage and video recorded for at least 10 min. Social (nose-to-nose, and anogenital sniffing) and non-social (exploring cage, sitting alone, and self-grooming) behaviors were analyzed by calculating the total time spent in seconds performing the analyzed behavior as described previously (Walsh et al., 2025). Investigators were blind to the mouse genotype during scoring analysis.

### 2.5 Mouse perfusion and tissue preparation

Transcardial perfusion, brain dissection and fixation by immersion overnight with 4% paraformaldehyde was performed as previously reported (Kim et al., 2023; Walsh et al., 2025). Following fixation and rinse with 1 × PBS, the brains were immersed in a 30% sucrose/PBS solution overnight at 4 °C, frozen in OCT, and cryosectioned into 45 μm and 50 μm floating sections.

### 2.6 Immunohistochemistry

Immunofluorescence experiments were performed as previously described (Walsh et al., 2025). The following primary antibodies were used: mouse anti-CALB (calbindin, 1:200, Sigma-Aldrich, #C9848), rabbit anti-IBA1 (1:200, Wako, #019-19741), goat anti-IBA1 (1:200, GeneTex, #GTX8979), rabbit anti-phosphorylated S6R (1:200, Cell Signaling, #4858), rabbit anti-pyruvate dehydrogenase E1 alpha (PDEH1 or PDH1A) (1:200, GeneTex, #GTX104015), and rat anti-CD107a (LAMP-1) (1:200, BioLegend, #121602), guineapig anti-VGLUT2 (1:200, Synaptic Systems, #135 404), mouse anti-neurofilament (1:200, BioLegend, # 837801), rabbit anti-pAMPK (1:200, Cell Signaling, #4060), rabbit anti-AMPK (1:200, GeneTex #GTX103487), rat anti mouse CD68-FA11 (1:200, Bio-Rad, MCA1957), and NeuroTrace (NT, Invitrogen). For mouse-derived primary antibodies, secondary antibody-only controls indicating the absence of neuronal detectable mouse-on-mouse background staining.

### 2.7 Microscopy image analysis

Images were acquired using a spinning disk confocal microscope (Yokogawa CSU-WI; Nikon) and analyzed with Bitplane Imaris™ as previously described (Walsh et al., 2025). Two to three confocal images per mouse were taken in the anterior lobule IV/V of the cerebellar vermis, which is involved in motor coordination and social processing (Chao et al., 2021). CALB, pAMPK, AMPK, pS6R, and NT were quantified using Surface renderings of PC somas. To quantify PDH1A and LAMP1 within CALB+ dendrites, a 300 × 500 μm region of the molecular layer was selected for CALB surface rendering, and dendritic immunoreactivity volumes were calculated as previously described (Walsh et al., 2025).

For NF, IBA1, and VGLUT2 quantification, Imaris was used to generate 3D surface renderings within a molecular layer region of interest (300 × 500 × 20 μm) and measure marker volume. The Filament tool was used to determine microglial process length and terminal points as previously described (Murray et al., 2025). IBA1+ microglia and CD68+ phagolysosomes were segmented using 3D surface rendering to quantify marker volume (Kavetsky et al., 2019). NeuroTrace-labeled PCs were counted manually using the ImageJ Cell Counter plugin (6 images/mouse), and soma diameter was measured in Imaris.

### 2.8 Statistical analysis

Analysis of data was performed using the GraphPad Prism software. Statistical significance was calculated using One-Way ANOVA for multiple comparisons with *post-hoc* Turkey Test. Also, Two-Way ANOVA with *post-hoc* Tukey's multiple comparisons between experimental groups when analyzing behaviors by genotype and sex. *p*-values are calculated by the GraphPad Prism software and significance was determined with *p*-values less than 0.05. Prior to statistical analyses, residual normality and homogeneity of variance were evaluated for behavioral datasets using GraphPad Prism 10. Residual distributions were assessed using normality tests, Q-Q plots, and residual diagnostics, and homogeneity of variance was evaluated using homoscedasticity plots. For behavioral measures that deviated from these assumptions, additional analyses were performed using log(Y+1)-transformed data to assess the robustness of the findings. These analyses yielded similar results and did not alter the interpretation of the data. Detailed statistical results, including F statistics, degrees of freedom, exact *p* values, and *post hoc* analyses, are provided in Supplementary Table S1.

## 3 Results

### 3.1 Voluntary running improves motor coordination in *Pten* cKO mice

At 5 month of age, the average distance per day (24 h) run by *Pten* cKO mice was calculated and compared to the average distance run by control mice that were also running since P21. We found that in both groups, the total running distance per day was variable between the mice in each group, still no differences were found between 5 month runners in the control or *Pten* cKO groups (Figure 1B), suggesting that lack of PTEN does not affect running performance.

Given that PC dysfunction affects motor coordination, the ladder rung walking task was used to determine the impact of voluntary running in the fine motor performance of *Pten* cKO mice (Figures 1C, D). As expected, 8 weeks *Pten* cKO SED mice had significantly more misses/slips in the ladder rung walking task than control mice; however, the number of misses/slips by *Pten* cKO RUN mice was significantly lower than *Pten* cKO SED and not different from controls (Figure 1C; *F*(2, 27) = 8.873, *p* = 0.001). Similar results were seen in mice at 7 months (Figure 1D; *F*(2,27) = 8.454, *p* = 0.0035). These results suggest that voluntary running improves fine motor function in both 8 weeks and 7 months old *Pten* cKO mice.

### 3.2 Effects of voluntary running in PTEN deficient Purkinje Cells' metabolic signaling and dendritic mitochondria volume

**Figure 2:**
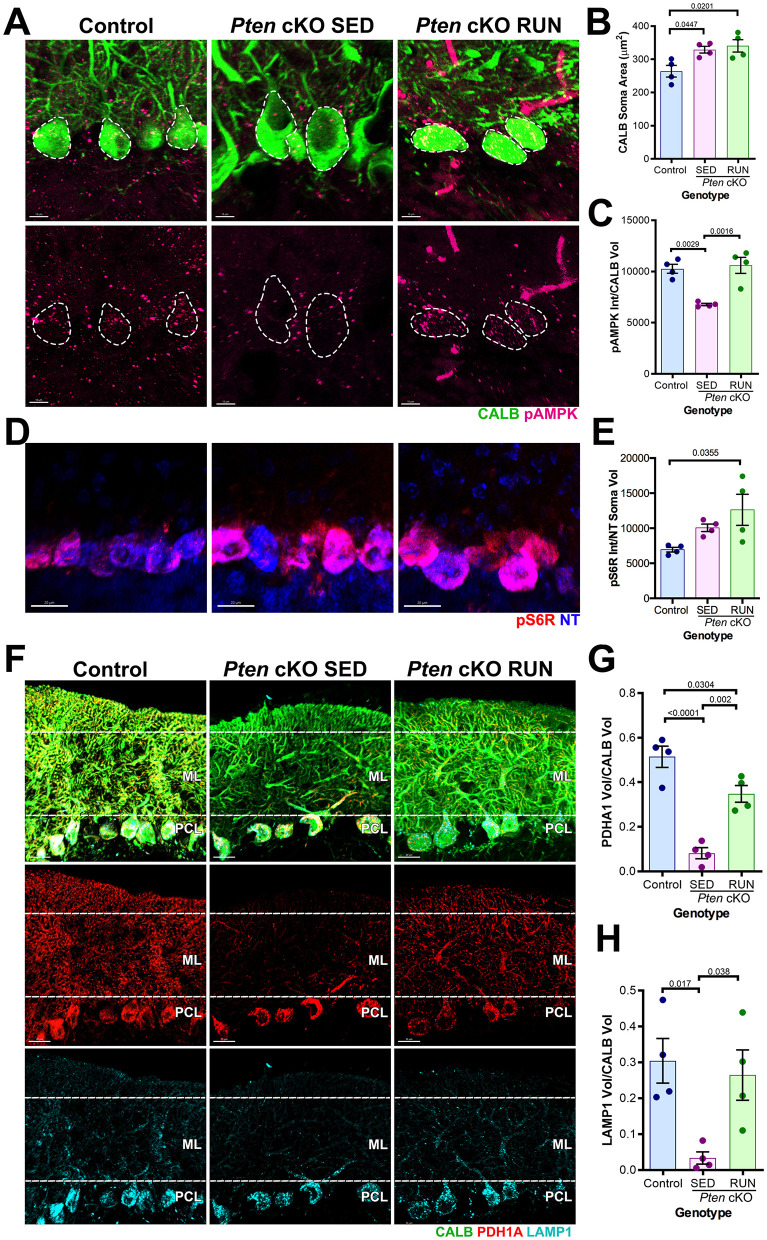
Voluntary running effects in the metabolic signaling of PTEN-deficient PCs. **(A)** CALB and pAMPK immunoreactivity in PCs from 8 week control, *Pten* cKO SED, and *Pten* cKO RUN mice. **(B)** Quantification of the CALB^+^ soma area of PCs. **(C)** Quantification of the ratio between the fluorescence intensity of pAMPK and CALB soma volume. **(D)** pS6R immunoreactivity and NT labeling in PCs from 8 week control, *Pten* cKO SED, and *Pten* cKO RUN mice. **(E)** Quantification of the ratio between pS6R fluorescence total intensity and NT soma volume in PCs. **(F)** Digitally segregated PDHE1 (mitochondria) and LAMP1 (lysosomes) immunoreactivity in CALB^+^ PC dendrites in control and experimental mice at 8wks. **(G)** Quantification of the ratio between dendritic PDHE1^+^ mitochondria volume and CALB^+^ PC dendrites' volume. **(H)** Quantification of the ratio of dendritic LAMP1^+^ lysosomes' volume and CALB^+^ PC dendrites' volume. Data are presented as mean ± SEM, *n* = 4 mice/genotype/age and 8–10 cells/mouse (B, C, and E). One-way ANOVA with *post-hoc* Tukey: **(B)** *p* = 0.017; **(C)** *p* = 0.001; **(E)** *p* = 0.045; **(G)** *p* < 0.0001; **(H)** *p* = 0.014. Scale bar: **(A)** 10 μm, **(D)** 20 μm and **(F)** 30 μm. ML, molecular layer; PCL, Purkinje cell layer.

Given that energetic stress and exercise are known to increase AMPK activation promoting mitophagy and mitochondria biogenesis (Reznick and Shulman, 2006), we investigated if voluntary running at 8 weeks could increase phosphorylated AMPK (pAMPK) levels in PC somas (Figures 2A–C). The soma area of CALB^+^ PCs was significantly increased in both *Pten* cKO SED and RUN PCs when compared to controls (Figure 2B; *F*(2, 9) = 6.624, *p* = 0.017). Still, voluntary running significantly increased pAMPK intensity/CALB soma volume in *Pten* cKO RUN mice compared to *Pten* cKO SED mice, whereas no difference was observed between *Pten* cKO RUN mice and controls (Figures 2A, C; *F*(2,9) = 16.30, *p* = 0.001). Analysis of non-phosphorylated AMPK immunoreactivity in PCs showed no significant differences between controls and experimental groups (Supplementary Figure 1).

We then quantified pS6R immunoreactivity, a downstream effector of mTORC1 signaling, after normalization to PC soma size using NeuroTrace (NT) labeling. Although pS6 immunoreactivity tended to be elevated in *Pten* cKO SED mice relative to controls (Figures 2D, E), this difference did not reach significance following *post hoc* testing. In contrast, runner mice exhibited significantly greater pS6 immunoreactivity than controls (Figures 2D, E; *F*(2,9) = 4.465, *p* = 0.045). These results suggest that voluntary running increases mTORC1 activation. Despite the pS6R findings, the ratio between the total volume of PDH1A^+^ mitochondria and the total volume of CALB^+^ PC dendrites was significantly increased in *Pten* cKO RUN mice compared to *Pten* cKO SED mice and but still lower than control levels (Figures 2F, G; *F*(2,9) = 33.24, *p* < 0.0001). Similarly, the ratio between the total volume of LAMP1^+^ lysosomes and the total volume of CALB^+^ PC dendrites was higher in *Pten* cKO RUN and control mice than in *Pten* cKO SED mice; no differences between *Pten* cKO RUN mice and controls were found (Figures 2F, H; *F*(2,9) = 7.045, *p* = 0.014). These findings suggest that voluntary running increases AMPK activation, lysosomal activity, and mitochondria density in PC dendrites.

### 3.3 Effects of voluntary running in social and non-social behaviors in *Pten* cKO mice

**Figure 3:**
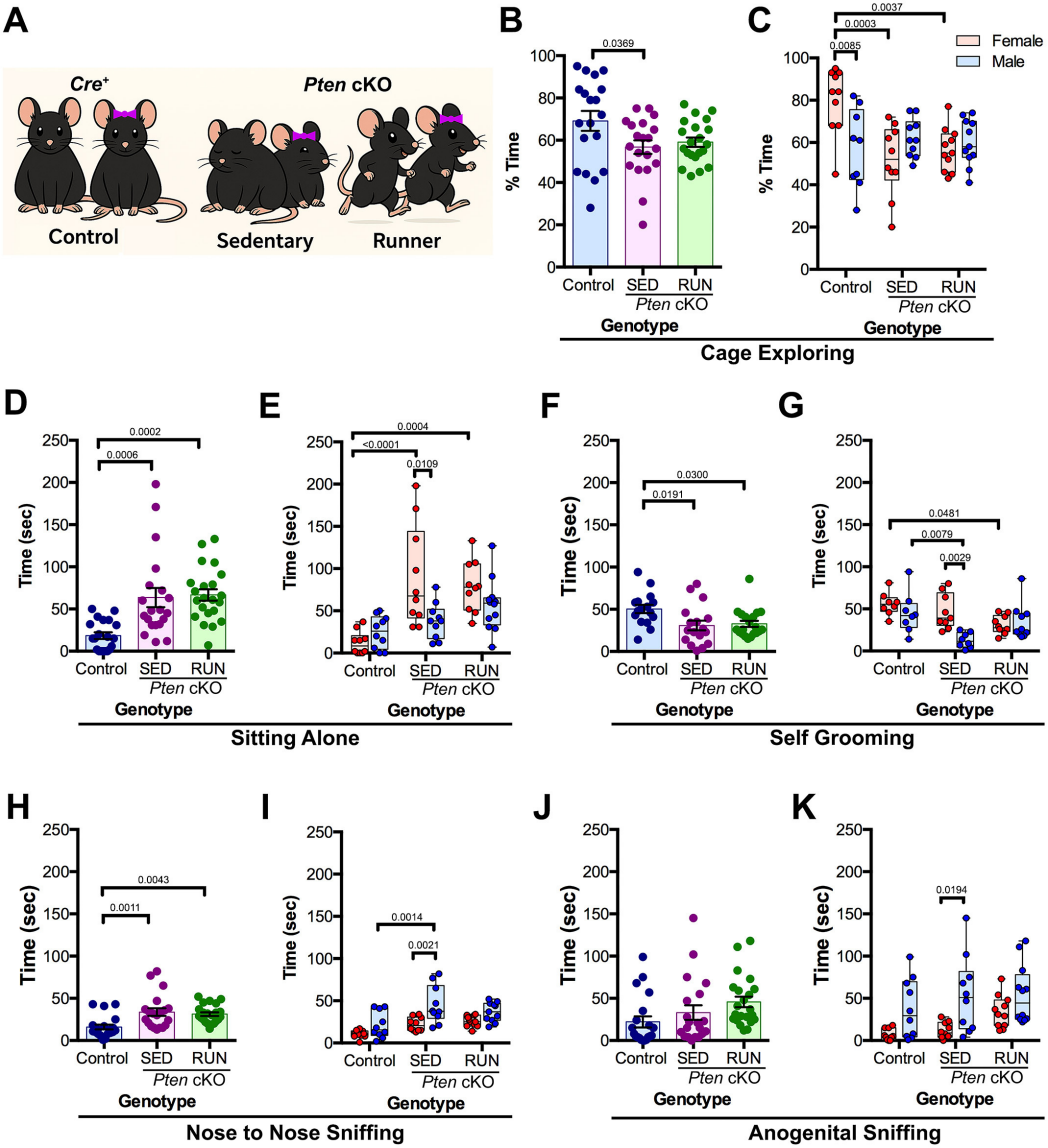
Assessing social and non-social behaviors. **(A)** Social and non-social behaviors were evaluated in heterosexual couples of control, *Pten* cKO SED, and *Pten* cKO RUN mice. **(B, C)** Percentage of time each mouse spent exploring the cage by genotype only **(B)** and by sex/genotype **(C)**. **(D, E)** Percentage of time each mouse spent sitting alone by genotype only **(D)** and by sex/genotype **(E)**. **(F, G)** Percentage of time each mouse spent on self-grooming by genotype only **(F)** and by sex/genotype **(G)**. **(H, I)** Percentage of time each mouse spent sniffing nose to nose by genotype only **(H)** and by sex/genotype **(I)**. **(J, K)** Percentage of time each mouse spent on anogenital sniffing by genotype only **(J)** and by sex/genotype **(K)**. Data presented as mean ± SEM, *n* = 8–11 mice/genotype/sex. Two-way ANOVA with *post-hoc* Tukey by Genotype: **(B, C)** *p* = 0.0202; **(D, E)** *p* < 0.0001; **(F, G)** *p* = 0.0029; **(H, I)** *p* < 0.0001; **(J, K)** *p* = 0.0526. Two-way ANOVA with *post-hoc* Tukey by Sex: **(C)** *p* = 0.4054; **(E)** *p* = 0.0185; **(G)** *p* = 0.0063; **(I)** *p* < 0.0001; **(K)** *p* < 0.0001.

Because heterozygous *Pten* mutations (Busch et al., 2019; Frazier, 2019) and cerebellar dysfunction are both associated with ASD (Crippa et al., 2016; Stoodley, 2016), we examined whether social and non-social behaviors were altered in 8 weeks *Pten* cKO mice and whether voluntary running could restore these behaviors. Control, *Pten* cKO SED, and *Pten* cKO RUN mice were paired with an opposite-sex mouse of the same genotype and experimental condition (Figure 3A). Behavioral interactions were analyzed by genotype and sex.

Two-way ANOVA revealed a significant interaction between sex and genotype/condition for cage exploration (*F*(2,55) = 7.86, *p* = 0.0010), as well as a significant main effect of genotype/condition (*F*(2,55) = 4.20, *p* = 0.0202), but no overall effect of sex (*F*(1, 55) = 0.70, *p* = 0.4054) (Figures 3B, C). *Post hoc* analyses showed that *Pten* cKO SED mice spent significantly less time exploring the cage than controls (Figure 3B). Sex-specific analyses indicated that this effect was primarily driven by females, as female controls explored the cage significantly more than female *Pten* cKO SED mice, whereas no significant differences were observed among males (Figure 3C). Notably, these differences were no longer observed in the *Pten* cKO RUN group, suggesting that voluntary running ameliorated the reduction in exploratory behavior.

Similarly, two-way ANOVA revealed a significant interaction between sex and genotype/condition for time spent sitting alone (*F*(2,56) = 4.86, *p* = 0.0113), together with significant main effects of genotype/condition (*F*(2,56) = 14.27, *p* < 0.0001) and sex (*F*(1,56) = 5.88, *p* = 0.0185) (Figures 3D, E). *Post hoc* comparisons demonstrated that both *Pten* cKO SED and RUN mice spent more time sitting alone than controls (Figure 3D). This effect was largely driven by females, as female *Pten* cKO SED and RUN mice spent significantly more time sitting alone than female controls, whereas no significant differences were detected among males (Figure 3E). In addition, female *Pten* cKO SED mice spent significantly more time sitting alone than male *Pten* cKO SED mice, a sex-dependent difference that was not observed in control or *Pten* cKO RUN groups.

Two-way ANOVA revealed a significant effect of genotype/condition on self-grooming behavior (*F*(2, 49) = 5.44, *p* = 0.0073) and a significant effect of sex (*F*(1,49) = 6.10, *p* = 0.0170). *Post hoc* analyses showed reduced self-grooming in *Pten* cKO SED and RUN mice compared with controls (Figure 3F). This reduction was most apparent in males, as male *Pten* cKO SED mice groomed significantly less than male controls (as previously reported by (Cupolillo et al., 2016) and by running females when compared to control females (Figure 3G).

For nose-to-nose interactions, two-way ANOVA revealed significant effects of genotype/condition (*F*(2, 56) = 11.42, *p* < 0.0001) and sex (*F*(1, 56) = 21.25, *p* < 0.0001), with no significant interaction between factors (*F*(2, 56) = 1.56, *p* = 0.2185) (Figures 3H, I). *Pten* cKO SED and RUN mice exhibited increased nose-to-nose interactions compared with controls (Figure 3H), while males displayed greater nose-to-nose interactions than females across groups. *Post hoc* analyses indicated that male *Pten* cKO SED mice showed increased interactions relative to male controls and *Pten* cKO SED females (Figure 3I). No significant interaction or genotype effect was observed for anogenital sniffing (Figures 3J, K). However, two-way ANOVA revealed a significant effect of sex (*F*(1, 57)=18.33, *p* < 0.0001), indicating that males exhibited greater anogenital interactions than females. Consistent with this finding, male *Pten* cKO SED mice showed increased anogenital interactions relative to *Pten* cKO SED females (Figure 3K).

Overall, these findings indicate that PTEN deficiency alters both social and non-social behaviors in a genotype and sex-dependent manner. Notably, voluntary running eliminated the sex-dependent differences observed in sedentary *Pten* cKO mice, suggesting a modulatory effect of exercise on behavioral outcomes.

### 3.4 Voluntary running reduces Purkinje cell loss in *Pten* cKO mice

**Figure 4:**
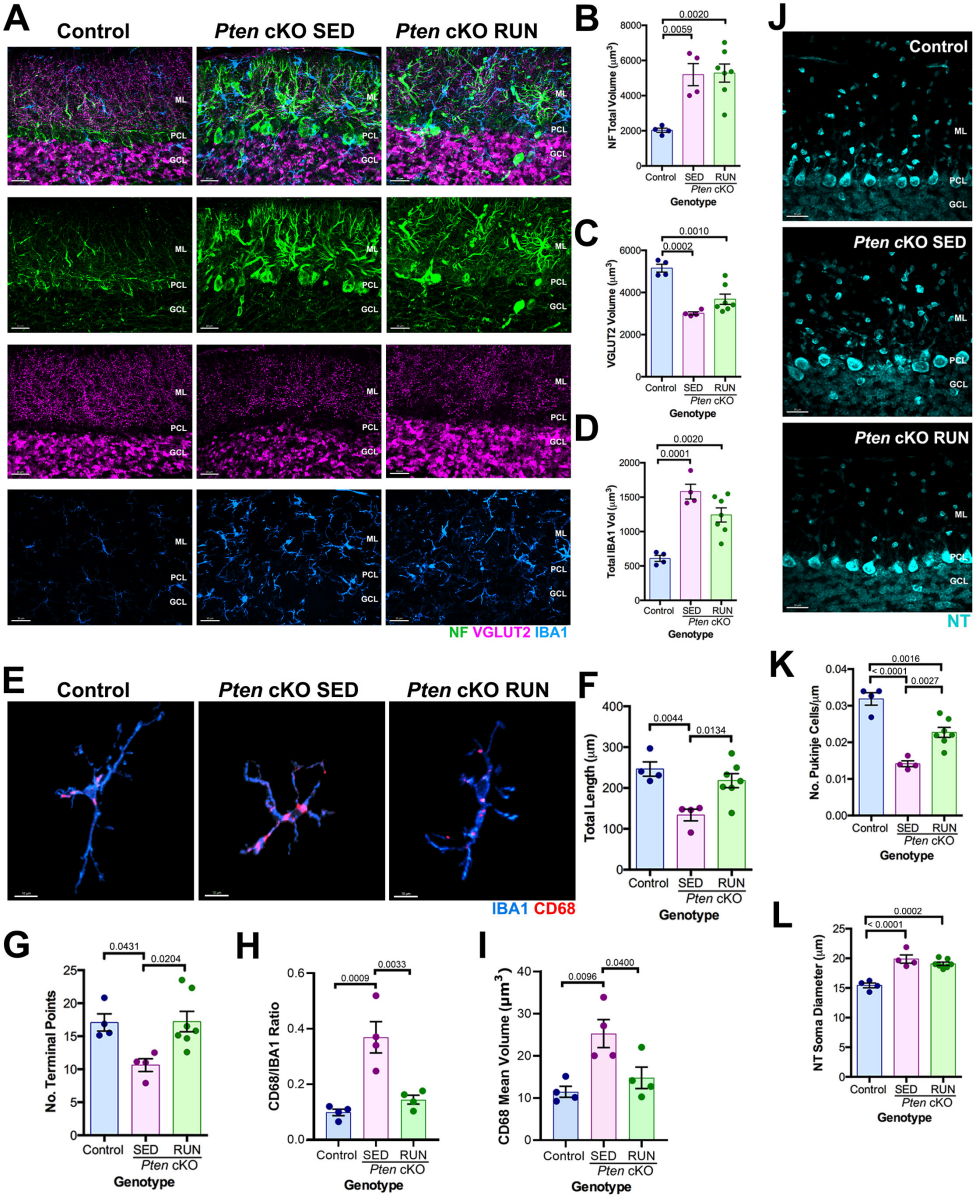
Voluntary running enhances PCs' survival despite pathological changes associated with PTEN deficiency. **(A)** Neurofilament, VGLUT2, and IBA1 immunoreactivity in the PCs and ML of 7 month contol, *Pten* cKO SED, and *Pten* cKO RUN mice. **(B–D)** Quantitative analysis of dendritic total volume of NF **(B)**, VGLUT2 **(C)**, and IBA1**(D)** in 7 month control and experimental mice. **(E)** Morphology of IBA1^+^ microglia and CD68^+^ phagolysosomes content per microglia in 7 month control and experimental mice. **(F, G)** Quantitative analyses of the total length **(F)** and the number of terminal points **(G)** of microglia from the ML. **(H, I)** Quantitative analyses of the ratio of CD68^+^ phagolysosomes per microglia **(H)** and the mean volume of CD68^+^ phagolysosomes **(I)**. **(J)** NeuroTrace staining of cerebellar sections showing changes in the number of PCs in 7 month control, *Pten* cKO SED, and *Pten* cKO RUN mice. **(K, L)** Quantitative analysis of the number of PCs per micrometer **(K)** and their soma diameter in control and experimental mice **(L)**. Data presented as mean ± SEM, for B, D, F, G, and J, K *n* = 4 (control and *Pten* cKO SED) or 7 mice (*Pten* cKO RUN), for H, I *n* = 4 mice/group (6 cells/mouse). One-way ANOVA with *post-hoc* Tukey: **(B)** *p* = 0.002; **(C)** *p* = 0.0002; **(D)** *p* = 0.0002; **(F)** *p* = 0.004; **(G)** *p* = 0.0181; **(H)** *p* = 0.0008; **(I)** *p* = 0.0096; **(K)** *p* < 0.0001; **(L)** *p* < 0.0001. Scale bar: (A and J) 30 μm, and **(E)** 10 μm. ML, molecular layer; PCL, Purkinje cell layer; GCL, Granule cell layer.

Dendritic hypertrophy and PC degeneration are pathological hallmarks found in *Pten* cKO mice over 6 months of age (Cupolillo et al., 2016; Walsh et al., 2025). To determine the long-term effect of voluntary running in NF^+^ dendritic hypertrophy, microglia activation, and PC degeneration, we studied adult 7 months control and experimental mice. A similar significant increase in NF^+^ hypertrophic dendrites was found in both *Pten* cKO SED and *Pten* cKO RUN mice compared to controls (Figures 4A, B; *F*(2, 12) = 11.37, *p* = 0.002). Also, *Pten* cKO SED and *Pten* cKO RUN PCs had significantly decreased VGLUT2^+^ presynaptic terminals compared to control mice (Figure 4A, C; *F*(2, 12) = 19.71, *p* = 0.0002). IBA1^+^ microglia total volume was significantly increased in both *Pten* cKO SED and *Pten* cKO RUN mice compared to controls and not significantly different between themselves (Figures 4A, D; *F*(2, 12) = 19.37, *p* = 0.0002). However, quantitative analysis of IBA1^+^ microglia morphology showed that control mice have significantly greater total length (*F*(2, 12) = 9.070, *p* = 0.004) and number of terminal points (*F*(2,12) = 5.711, *p* = 0.0181) compared to *Pten* cKO SED but similar to *Pten* cKO RUN mice (Figures 4E–G), suggesting a reduced activated phenotype in microglia from *Pten* cKO RUN mice. In fact, a significant higher total volume of CD68^+^ phagolysosomes per IBA1^+^ microglia was found only in *Pten* cKO SED mice (*F*(2, 9) = 17.62, *p* = 0.0008), and the mean volume of these CD68^+^ phagolysosomes was also larger in these mice when compared to the other two groups (Figures 4E, H, I and Supplementary Figure 2; *F*(2, 9) = 8.135, *p* = 0.0096), supporting an activated phagocytic microglial phenotype in *Pten* cKO SED but not in *Pten* cKO RUN mice.

Lastly, NeuroTrace (NT) labeling of PC somas, revealed a significantly decreased number of PCs and increased soma diameter in *Pten* cKO SED and *Pten* cKO RUN mice compared to controls (Figures 4J–L; *F*(2,12) = 31.14, *p* < 0.0001). Nonetheless, long-term running appears to attenuate PC loss associated with PTEN deficiency, as *Pten* cKO RUN mice exhibited significantly greater PC numbers than *Pten* cKO SED mice (Figures 4J–L). Although direct measures of apoptosis were not assessed, the preservation of PCs in the running group, together with the reduction in microglial CD68-positive phagolysosomes, suggests that voluntary running mitigates neurodegenerative pathology in *Pten* cKO mice.

## 4 Discussion

The PTEN–mTORC1 signaling axis is a key regulator of neuronal growth and metabolic homeostasis. PTEN negatively regulates PI3K/Akt/mTOR signaling, thereby balancing anabolic and catabolic processes. Loss of PTEN results in sustained mTORC1 activation, promoting cellular overgrowth while impairing energy balance and metabolic function (Carracedo and Pandolfi, 2008). We previously demonstrated that PTEN deletion in PCs leads to reduced AMPK activation and decreased mitochondrial and lysosomal content, suggesting impaired energy sensing and metabolic stress (Walsh et al., 2025). Because physical exercise is a known activator of AMPK (Reznick and Shulman, 2006; Huang et al., 2019), we investigated whether voluntary running could restore metabolic balance in PTEN-deficient PCs.

Variability in daily running distance (24 h) ranged from 0.5 to 10 km per day and was comparable between control and *Pten* cKO mice, consistent with previously reported values in mice (Goh and Ladiges, 2015). Importantly, voluntary running significantly improved performance of *Pten* cKO mice in the horizontal ladder rung task, a behavioral assay that assesses motor coordination, skilled walking, and limb placement (Metz and Whishaw, 2009). Because these functions are largely regulated by cerebellar circuits (Morton and Bastian, 2004), these findings suggest that voluntary running induces functional improvements in the cerebellum of *Pten* cKO mice.

A growing body of evidence demonstrates that physical exercise increases AMPK activation across multiple tissues, including the hippocampus (Spaulding and Yan, 2022). In hippocampal neurons, exercise-induced AMPK activation has been shown to enhance mitochondrial protein expression (Bayod et al., 2015) and increase lysosomal function in the brain (Huang et al., 2019). Consistent with these findings, we observed increased pAMPK immunoreactivity in PCs from PTEN-deficient runner mice.

Interestingly, voluntary running further amplified the elevated pS6R immunoreactivity associated with PTEN deficiency, indicating increased mTORC1 activity. Although not the primary goal of our study, previous reports have also shown that exercise can activate mTORC1 signaling in the brain (Lloyd et al., 2017; Chen et al., 2019) in response to the expression of Brain derived neurotrophic factor (BDNF) in PCs (Cook et al., 2022), promoting neuroplasticity (Tang et al., 2025). This sustained mTORC1 activation in PTEN-deficient PCs from runner mice may contribute to pathological features that were not rescued by exercise, including neurofilament accumulation, somatic and dendritic hypertrophy, and loss of VGLUT2^+^ presynaptic terminals from climbing fibers.

Hyperactivation of mTORC1 by PI3K/AKT promotes cellular overgrowth by sustaining anabolic processes that also deplete cellular ATP levels. Under normal conditions, ATP depletion activates AMPK, which in turn inhibits mTORC1 through the activation of TSC2 (tuberous sclerosis complex), stimulating catabolic pathways such as autophagy and mitophagy (Liu and Sabatini, 2020). However, running-induced pAMPK accumulation failed to inhibit mTORC1 activation in PTEN-deficient PCs. This resistance likely stems from AKT's ability to directly activate mTORC1, bypassing the classical AMPK/TSC2 axis to maintain downstream signaling (Wiza et al., 2012).

Activated AMPK enhances PGC-1α (Peroxisome proliferator-activated receptor gamma coactivator 1-alpha) expression and activity, promoting the transcription of nuclear and mitochondrial genes required for mitochondrial biogenesis (Reznick and Shulman, 2006; Malik et al., 2023). In contrast, persistent mTORC1 activation drives mitochondrial fragmentation through 4E-BP-mediated translation of mitochondrial fission factor 1 (MTFP1) (Morita et al., 2017), suppresses AMPK activity (Ling et al., 2020), and inhibits autophagy (Bartolome et al., 2017). These effects lead to the accumulation of damaged and dysfunctional mitochondria. Impaired mitophagy subsequently disrupts mitochondrial turnover, contributing to metabolic decline, neurodegeneration, and aging (Palikaras et al., 2015; Cianfanelli et al., 2025). Despite the elevated mTORC1 activity observed in PTEN-deficient PCs from runner mice, both mitochondrial and lysosomal volumes in dendrites were significantly increased. This suggests that AMPK activation induced by voluntary running may partially counteract the detrimental effects of mTORC1 hyperactivation. Supporting this interpretation, previous studies have shown that exercise promotes mitochondrial biogenesis (Hood and Saleem, 2007) and reduces mitochondrial fission in muscle (Lin et al., 2024), largely through AMPK-dependent mechanisms. Together, these findings support a model in which exercise-induced AMPK activation restores key aspects of metabolic homeostasis in PTEN-deficient neurons, even in the presence of persistent mTORC1 signaling.

Behavioral analyses revealed that PTEN deficiency alters both social and non-social behaviors, with notable sex-dependent differences. While voluntary running did not fully normalize these behaviors, it eliminated sex-dependent effects observed in sedentary *Pten* cKO mice. The increased social investigation observed in *Pten* cKO mice may reflect impaired behavioral inhibition rather than enhanced sociability. PCs provide the sole inhibitory output of the cerebellar cortex, and disruption of cerebellar circuits has been implicated in deficits in behavioral flexibility, inhibitory control, and social regulation (Carta et al., 2019). Thus, the increased nose-to-nose and anogenital interactions observed in *Pten* cKO mice may represent excessive or poorly modulated social behaviors resulting from reduced cerebellar control over downstream reward and executive circuits. Consistent with this interpretation, cerebellar activity regulates autism-associated behaviors through functional connections with the medial prefrontal cortex and other limbic regions involved in social decision-making (Kelly et al., 2020). Voluntary running reduced several of these behavioral abnormalities, suggesting that exercise may partially restore inhibitory control over maladaptive social behaviors. This interpretation is further supported by clinical studies demonstrating beneficial effects of exercise on social communication, emotional regulation, and repetitive behaviors in individuals with autism spectrum disorder (Wang et al., 2025).

PC survival during pathological conditions is enhanced by exercise (Yun et al., 2014; Jin et al., 2023). The reduced number and size of CD68-positive phagolysosomes observed in *Pten* cKO RUN mice suggest decreased microglial phagocytic activity. This finding is consistent with the preservation of PCs in the running group and indicates that voluntary exercise attenuates pathological processes associated with PTEN deficiency.

Several limitations should be considered. Although voluntary running was associated with increased PC numbers and reduced microglial phagocytic activity, direct measures of apoptosis were not successfully validated, and therefore future studies are needed to determine whether exercise directly promotes PC survival. In addition, the increased social investigation observed in *Pten* cKO mice may reflect impaired behavioral inhibition rather than enhanced sociability. Future studies employing behavioral paradigms specifically designed to assess impulsivity, compulsivity, and inhibitory control will be necessary to further define the contribution of PC dysfunction to these behavioral abnormalities. Finally, the limited number of biological replicates in some histological analyses warrants cautious interpretation and validation in larger cohorts. Together, these findings demonstrate that voluntary running partially ameliorates behavioral and cerebellar abnormalities associated with PTEN deficiency.

## Data Availability

The raw data supporting the conclusions of this article will be made available by the authors, without undue reservation.
