# Supplementary material for: Voluntary running reduces loss of PTEN-deficient Purkinje cells, modulates their metabolic signaling, and improves motor deficits in mice
Source: Front Cell Neurosci. 2026 Jul 22;20:1865302. doi: 10.3389/fncel.2026.1865302 (PMC13437335; doi:10.3389/fncel.2026.1865302)
Supplement: Supplementary file 1 [file Supplementary_File_1.docx]

Supplementary Material

# 1 Supplementary Figures and Tables

## 1.1 Supplementary Figures

**Figure S1.**

**Supplementary Figure 1.** AMPK immunoreactivity in Purkinje cells from Control, *Pten* cKO sedentary, and *Pten* cKO running mice. Quantitative analysis of the ratio between AMPK total intensity and the NeuroTrace labeled PC soma shows no statistically significant differences between groups. Data presented as mean ± SEM, *n* = 4 mice per group. Scale bar: 20 μm.

**Figure S2.**

**Supplementary Figure 2.** IBA1 and CD68 in the cerebellar molecular layer from Control, *Pten* cKO sedentary, and *Pten* cKO running mice. CD68^+^ phagolysosomes are significantly increased in microglia from *Pten* cKO sedentary mice when compared to control and *Pten* cKO running mice. Scale bar: 20 μm.

## 1.2 Supplementary Tables

**Supplementary Table S1. Summary of statistical analyses.**
